# Pick up and dispose of pollutants from water via temperature-responsive micellar copolymers on magnetite nanorobots

**DOI:** 10.1038/s41467-022-28406-5

**Published:** 2022-03-01

**Authors:** Jayraj V. Vaghasiya, Carmen C. Mayorga-Martinez, Stanislava Matějková, Martin Pumera

**Affiliations:** 1 Center for Advanced Functional Nanorobots, Department of Inorganic Chemistry, Faculty of Chemical Technology, University of Chemistry and Technology Prague, Technická 5, 166 28, Prague, 6 Czech Republic; 2 Central Analytical Laboratory, Institute of Organic Chemistry and Biochemistry of the Academy of Sciences of the Czech Republic, 166 10, Prague, 6 Czech Republic; 3 Department of Chemical and Biomolecular Engineering, Yonsei University, 50 Yonseiro, Seodaemun-gu, Seoul, 03722 Korea; 4 Department of Medical Research, China Medical University Hospital, China Medical University, No. 91 Hsueh-Shih Road, Taichung, 40402 Taiwan; 5 Center for Nanorobotics and Machine Intelligence, Dept. of Food Technology, Mendel University, Zemedelska 1, Brno, 613 00 Czech Republic; 6 Future Energy and Innovation Lab, Central European Institute of Technology, Brno University of Technology, Purkyňova 123, Brno, 612 00 Czech Republic

**Keywords:** Nanocomposites, Materials for devices, Pollution remediation

## Abstract

Nano/micromotor technology is evolving as an effective method for water treatment applications in comparison to existing static mechanisms. The dynamic nature of the nano/micromotor particles enable faster mass transport and a uniform mixing ensuring an improved pollutant degradation and removal. Here we develop thermosensitive magnetic nanorobots (TM nanorobots) consisting of a pluronic tri-block copolymer (PTBC) that functions as hands for pollutant removal. These TM nanorobots are incorporated with iron oxide (Fe_3_O_4_) nanoparticles as an active material to enable magnetic propulsion. The pickup and disposal of toxic pollutants are monitored by intermicellar agglomeration and separation of PTBC at different temperatures. The as-prepared TM nanorobots show excellent arsenic and atrazine removal efficiency. Furthermore, the adsorbed toxic contaminants on the TM nanorobots can be disposed by a simple cooling process and exhibit good recovery retention after multiple reuse cycles. This combination of temperature sensitive aggregation/separation coupled with magnetic propulsion opens a plethora of opportunities in the applicability of nanorobots in water treatment and targeted pollutant removal approaches.

## Abstract (web summary)

Pesticide and heavy metal pollution in water can cause environmental and public health issues. Here, the authors report thermoresponsive magnetic nanorobots that can efficiently pick up and dispose of pollutants from water by adjusting the water temperature.

## Introduction

Artificial nano/micromotors are an emerging technology for remote-controlled mobility and the performance of complex tasks on the nano/microscale. They have been shown to be not only desirable for applications in the environmental field for pollutant removal, but also in the medical-field applications using external stimuli such as magnetic, ultrasound, and light sources^1–4^. Various kinds of light-powered nano-/micromotors have been described for cleaning polluted water, but further research is needed, especially in the areas of propulsion, stability, and directionality^5,6^. However, magnetically driven nano-/micromotors have drawn significant attention in recent years due to their capability to accelerate with great accuracy on a nano- and micrometer-length scale without the need for chemical fuel^7–11^. Furthermore, it has the benefits of easy manufacturing, low cost, and long-lasting motion, which hold promise for eco-friendly nano/micromotors to replace hazardous solvents (e.g., hydrogen peroxide) and noble-metal catalysts (e.g., Pt, Au, and Ag). Recently, various kinds of magnetic nano-/micromotors have demonstrated tremendous and exciting outcomes in the fields of biomedicine (e.g., cargo loading/release, therapy)^12–18^ and water treatment (e.g., removal of toxic organic–inorganic pollutants)^3,5,19–25^. Other applications include self-powered porous spore@Fe_3_O_4_ biohybrid micromotors^20^ for the removal of toxic lead ions; mesoporous CoNi@Pt nanomotors, T/Fe/Cr micromotors and Fe_3_O_4_ nanoparticles are utilized for degradation of organic pollutants^26–28^; SW-Fe_2_O_3_/MnO_2_ micromotors used for oxidation of anthraquinone dyes/chlorophenols^29^; and MnFe_2_O_4_/oleic acid micromotors and Mg/Ti/Ni/Au Janus micromotors for oil removal^22,30^. However, the majority of magnetic nano/micromotors used in water remediation rely on a metal (Pt, Au) catalyst that causes a lot of problems during practical applications; a long-time metal-catalyst contact with aqueous media is easily oxidized and restricts the lifespan of nano-/micromotors. Therefore, it is vital to explore low-cost, environmentally friendly, and reusable nano/micromotors for the pickup and disposal of pollutants, especially arsenic metal ions and organic pesticides, which are incredibly harmful to the atmosphere and biosphere.

In context, if the surface of autonomous nano-/micromotors could be modified with polymeric materials, then synthesis expenses would be reduced, remediation efficiency would be improved, and above all, adverse effects would be diminished. For instance, T. Hou et al.^31^ used a copolymer of aspartic acid and cysteine-templated Ni–Pt micromotors for the elimination of heavy-metal ions such as lead (Pb^+2^), cadmium (Cd^+2^), and mercury (Hg^+2^). Janus stearic acid-modified polyvinyl alcohol foam motors were used for oil absorption^32^. Moreover, poly(3,4-ethylenedioxythiophene) polystyrene sulfonate (PEDOT:PSS) with metal (Pt or Au) catalyst-based micromotors was employed for the selective pickup of heavy-metal ions, bacteria, and oil droplets in a realistic water environment^5,33–35^. However, the manufacturing process of these polymeric nano-/micromotors is complicated and they exhibit poor mechanical characteristics, for example, their reusability and application as an adsorbent in water are restricted.

Here, we show a facile method for the preparation of thermosensitive magnetic nanorobots (TM nanorobots) using PTBC and magnetite nanoparticles (Fe_3_O_4_) to pick up and dispose of pollutants efficiently. PTBC is a family of environmentally friendly and temperature-responsive micellar copolymers in water suspension. We report that the self-assembly of the PTBC–TM nanorobots takes place when the temperature of the solution increases from 5 °C to 25 °C. During this self-assembly time, pollutants such as atrazine and arsenic are picked up by the TM nanorobots. In addition, we also demonstrate that the pollutants adsorbed on the nanorobots can be discarded and the nanorobots can be retrieved through a simple cooling step that enables recycling and reuse for further cleaning applications. These new TM nanorobots show excellent capability for the pickup and disposal of toxic contaminants in water and create new opportunities for targeted and complex environmental remediation.

## Results and discussion

**Fig. 1:**
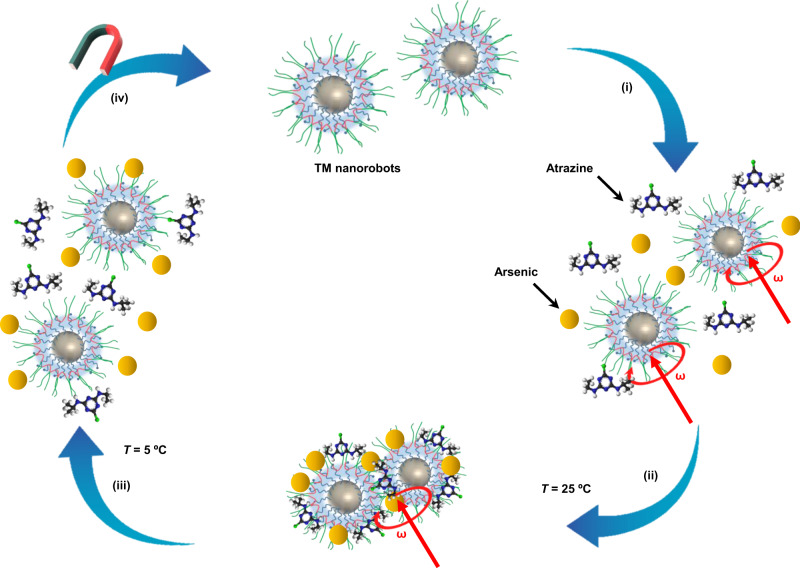
Formulation of TM nanorobots for water-treatment applications. (i) TM nanorobots walking in polluted water under a transversal rotating magnetic field (ω), (ii) pollutants are picked up by the TM nanorobots via intermicellar aggregation at ambient temperature (T) under a transversal rotating magnetic field, (iii) separation of TM nanorobots to dispose of the pollutants from PTBC matrix at low temperature, and (iv) TM nanorobots recovered by using an external magnet.

Thermomagnetic nanorobots are prepared using magnetite nanoparticles (Fe_3_O_4_) coated by thermoresponsive PTBC micellar polymer. Prior to describing the synthesis process and examining the characterizations of TM nanorobots, it is essential to discuss the concept and work function of TM nanorobots for the pickup and disposal of toxic pollutants in water. The experimental design and mechanism of the proposed TM nanorobots, composed of a hydrophobic core (poly(propylene oxide) (PPO)) and thermoresponsive hydrophilic (poly(ethylene oxide), (PEO)) shell, are schematically shown in Fig. 1.

The hydroxyl-terminated PTBCs on the surface of Fe_3_O_4_ nanoparticles have an amphiphilic nature driven by the intensive and partial interaction of PEO and PPO segments with water molecules, respectively^36–38^. Therefore, PTBC shows a reversible temperature-sensitive phase transformation in water: it would be easily dispersed in water medium below their critical micelle temperature (CMT), but precipitates above the CMT^39–42^. Although the PTBCs are now integrated, it is feasible that micellization-like self-assembly can occur on TM nanorobots’ surfaces. The copolymer chains are completely expanded by engaging with water at lower CMT and then the copolymer shell is accessible (step-i).

When the temperature is raised (~25 °C) above the CMT, the PTBC dehydration and stronger interactions between the polymer blocks themselves (e.g., PEO/PEO and PPO/PPO) lead pollutants to be trapped in the PTBC matrix. At the same time, when a transversal rotating magnetic field is applied, the interaction between the polymer blocks on the TM nanorobots is more efficient as is evident by the strong aggregation of TM nanorobots (step-ii). Due to the apparent magnetic sensitivity of Fe_3_O_4_, the TM nanorobots could be driven to the desired destination by applying a magnetic field. Afterward, the PTBC matrix is entirely expanded by interacting with water at low temperature (~5 °C), thus opening the PTBC shell and allowing the pollutant to be retrieved (step-iii). After the disposal procedure, TM nanorobots can be simply recovered by using an external magnet and ready to use for the next cycle (step-iv). Assessments of the performance of each step and their characterizations are listed in the next section. In addition, PTBCs not only provide a thermosensitive property for the aggregation and separation of nanorobots but also minimize surface oxidation of the nanoparticles^43,44^ and increase stability toward environmental remediation.

**Fig. 2:**
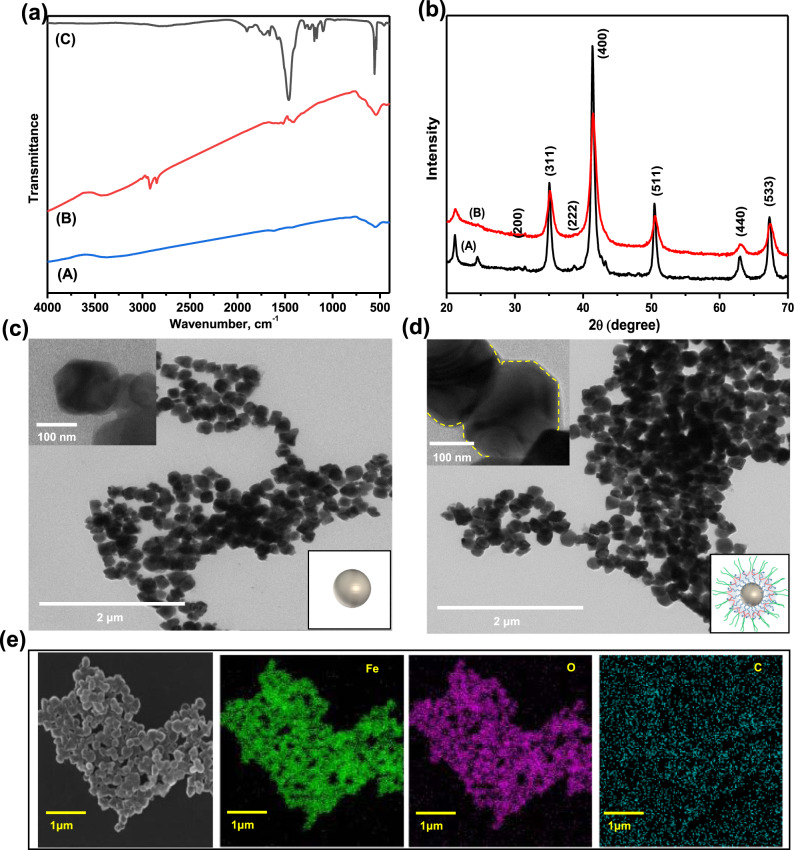
Structural and morphological characterizations of TM nanorobots. **(a)** FTIR spectra of pristine Fe_3_O_4_ nanoparticles (A), oleic acid-functionalized Fe_3_O_4_ nanoparticles (B), and TM nanorobots (C) in the range of 4000–400 cm^−1^. (**b)** XRD pattern of pristine Fe_3_O_4_ nanoparticles (A) and TM nanorobots (B). TEM images of (**c**) pristine Fe_3_O_4_ nanoparticles and (**d**) TM nanorobots (inset shows magnified TEM images), (**e**) EDS mapping of TM nanorobots.

The detailed fabrication method of TM nanorobots by sequential immobilized oleic acid and PTBC on Fe_3_O_4_ nanoparticles is mentioned in the experimental section. Briefly, Fe_3_O_4_ nanoparticles were synthesized by coprecipitation of iron–chloride salts (FeCl_3_ 6H_2_O and FeCl_2_ 4H_2_O) and hydroxyl functional groups generated at their surface were substituted with oleic acid to form excess carboxyl groups and achieve an anionic surface^45–47^. The obtained oleic acid–functionalized Fe_3_O_4_ nanoparticles are hydrophobic and not dispersible well in an aqueous solution. However, this formulation easily allows a hydrophilic (PEO)/hydrophobic (PPO) block copolymer to attach onto the oleic acid-functionalized Fe_3_O_4_ nanoparticle core (via Van der Waals interactions^48^) for aqueous dispersion. Supplementary Fig. 1 schematically demonstrates a possible reaction mechanism for Fe_3_O_4_ nanoparticle modification. Successful copolymer immobilization on Fe_3_O_4_ nanoparticles was validated by FTIR spectroscopy. FTIR spectra of pure Fe_3_O_4_ nanoparticles, oleic acid-decorated Fe_3_O_4_ nanoparticles, and TM nanorobots are shown in Fig. 2a. FTIR spectra of oleic acid-functionalized Fe_3_O_4_ nanoparticles express 2933 and 2834 cm^−1^ peaks that align to –CH_2_ asymmetric and symmetric stretching, respectively. The peaks at 1439 and 1531 cm^−1^ correspond to asymmetric *ν*_*s*_(–COO^-^) and symmetric *ν*_*s*_(–COO^-^) stretch vibration, respectively. These findings show that oleic acid is chemisorbed onto the Fe_3_O_4_ nanoparticles as a carboxylate. The peak observed at 565 cm^−1^ is attributed to Fe_3_O_4_ nanoparticles. The intense peak at 1464 cm^−1^ and broad peaks between 1000 and 1200 cm^−1^ in the spectrum (Fig. 2a(C)) indicate C–O stretching vibration, confirming the availability of PTBC. The functionalization of Fe_3_O_4_ nanoparticles should occur only at its surface in order to retain the state of the bulk counterparts, such as crystalline phase, and crystallinity should be unchanged. If the chemical reaction modifies the state of the bulk properties, then the resulting TM nanorobots lose their inherent properties. Therefore, assurance of PTBC functionalization on the surface of Fe_3_O_4_ nanoparticles and the retention of crystalline characteristics are crucial to fabrication. The crystal structure of the synthesized Fe_3_O_4_ nanoparticles and TM nanorobots was identified by X-ray powder-diffraction (XRD) measurement.

The recorded XRD pattern of pristine Fe_3_O_4_ nanoparticles and TM nanorobots is depicted in Fig. 2b. As we can see, strong and intense peaks suggest that the prepared Fe_3_O_4_ nanoparticles are crystalline nature. The Bragg’s diffraction peaks observed at 2θ = 31.47, 35.05, 38.66, 43.28, 50.43, 62.83, and 74.10 attributing to (220), (311), (222), (400), (511), (440), and (533) crystal planes of Fe_3_O_4_ nanoparticles are not distinct from those of TM nanorobots. Such a result indicates that while copolymer immobilization did not change their crystalline structure, the intensity of specified peaks declined by a real value as a consequence of PTBC functionalization on Fe_3_O_4_ nanoparticles (or TM nanorobots). The morphology of the as-prepared Fe_3_O_4_ nanoparticles and TM nanorobots was analyzed by transmission electron microscopy (TEM). As shown in Fig. 2c, d, the Fe_3_O_4_ nanoparticles and TM nanorobots had nonregular hexagonal morphology with relatively uniform shape and size (>200 ± 25 nm). Inset of Fig. 2d, we can see the distinct PTBC wrapping layer on the Fe_3_O_4_ nanoparticles’ surface. The energy-dispersive X-ray spectrometry (EDS) mapping images of TM nanorobots confirm that the primary constituent elements are Fe, C, and O (Fig. 2e and Supplementary Fig. 2). The PTBC structure comprises a majority of O and C. The PTBC surrounding of magnetite nanoparticles’ core was not mapped.

The effect of temperature on TM nanorobot morphology was examined using scanning transmission electron microscopy (STEM) and EDS mapping, and the images are displayed in Supplementary Fig. 3. At 25 °C above the critical micelle temperature, most of the particles are clearly visible in the aggregate state (Supplementary Fig. 3a). Although they are uniformly scattered at 5 °C, the presence of a few aggregates cannot be fully ruled out at this temperature (Supplementary Fig. 3c). EDS was used to characterize these STEM images, which validated the composition and distribution of the elements in TM nanorobots (Supplementary Fig. 3b, d). When comparing the EDS maps, we noticed that the amount of Fe and O reduces at low temperatures. This result reveals that the separation of TM nanorobots occurs when the temperature goes below 5 °C. Furthermore, we analyzed the surface charge of the oleic acid-treated Fe_3_O_4_ nanoparticles and TM nanorobots in water through a zeta-potential analyzer. The zeta potential (ζ) of oleic acid-coated Fe_3_O_4_ nanoparticles, −7.178 mV, changed to −4.445 mV after loading of PTBC. This finding indicates that TM nanorobots can be utilized for the elimination of positively charged organic and inorganic contaminants.

**Fig. 3:**
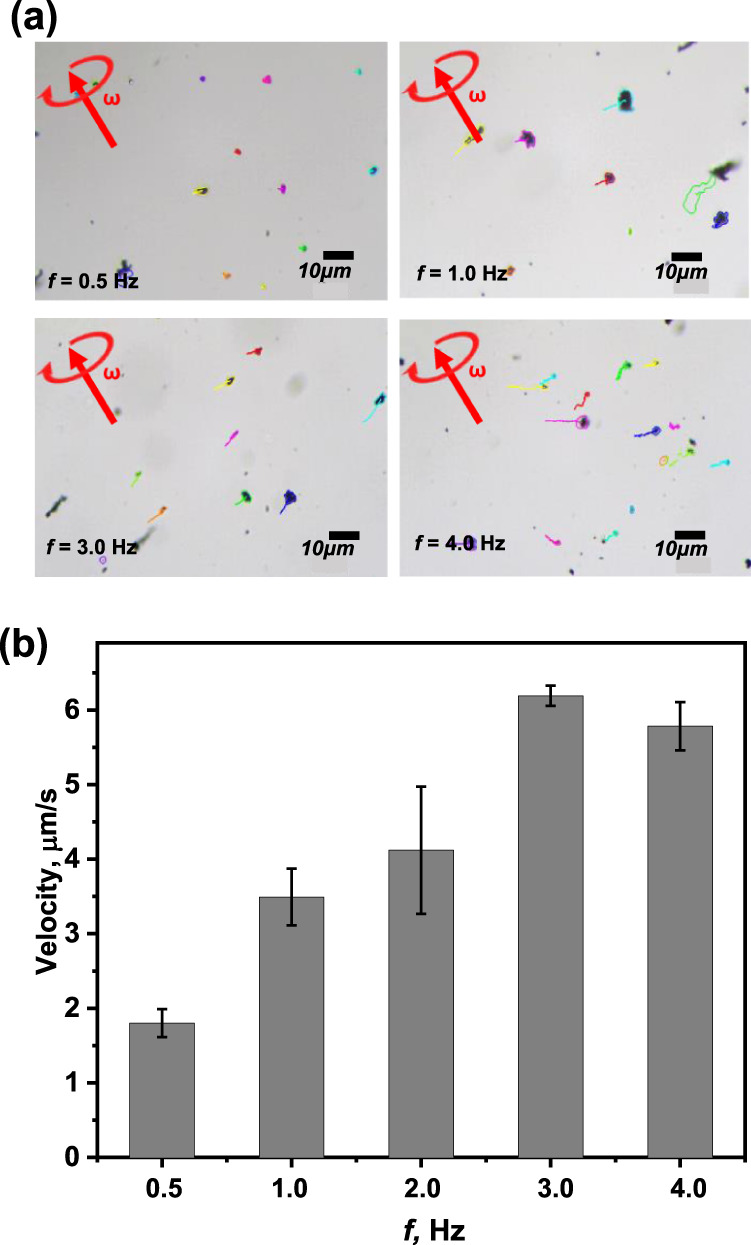
Tracking of TM nanorobots. (**a**) optical images highlight the magnetic work function of the TM nanorobots using the transversal rotating magnetic frequency range of 0.5–4 Hz and (**b**) quantitative velocity of the TM nanorobots as a function of rotating magnetic-field frequency. All velocity data are summed for 30 TM nanorobots under the same propulsion conditions. The error bars represent standard deviation based on three measurements.

Following the systematic and morphology characterizations of TM nanorobots, their velocities under transversal rotating magnetic field with amplitude of 3 mT and frequency range of 0.5–4.0 Hz were evaluated and are shown in Fig. 3a. Figure 3b illustrates the increasing speed of TM nanorobots in response to rising input frequency of the applied magnetic field. The velocity of TM nanorobots was observed at around 1.78 µm s^−1^ at the low frequency of 0.5 Hz. When using a frequency of 3 Hz, the TM nanorobots achieved the highest velocity of 6.21 µm s^−1^, whereas constant speed was recorded at higher frequencies. Although the input frequency is elevated beyond the move-out frequency (here >4 Hz), the fluidic drag reaches the highest accessible magnetic torque, adding to the decline in velocity as the nanorobots move out-of-sync with the applied magnetic environment^8,49^.

**Fig. 4:**
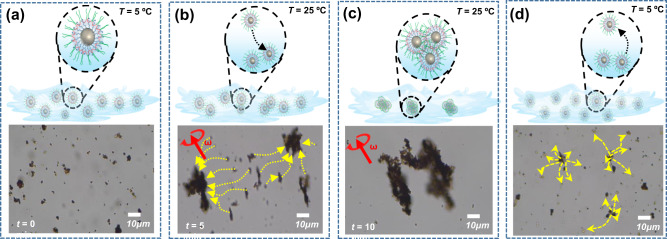
Schematic representation of TM nanorobot motion during pickup and disposing of toxic pollutants under a transversal rotating magnetic field. **(a)** scattered TM nanorobots, (**b**) begin accumulating of TM nanorobots with their toxic payloads, (**c**) aggregated TM nanorobots, and (**d**) dispersion of TM nanorobots and toxic pollutants’ disposal at low temperature.

Further, to examine whether the self-assembly, intermicellar aggregation, toxic pollutant pickup, and disposal operations were performed at different temperatures (~5 °C to ~25 °C) with optimized rotational magnetic frequency (3 Hz, 3 mT). At low temperature, the TM nanorobots are well dispersed in polluted-water media (Fig. 4a and panel a in Supplementary Movie 1). When a magnetic field is implemented, TM nanorobots move to the desired area and begin to accumulate (Fig. 4b). Upon reaching room temperature, the TM nanorobots begin to self-assemble by intermicellar aggregation, trapping the pollutants inside the PTBC matrix (Fig. 4c and panel b in Supplementary Movie 1). Finally, once the TM nanorobots are cooled, they have dispersed again (Fig. 4d and panel c in Supplementary Movie 1), disposing of pollutants into the solution.

By taking advantage of the temperature-responsive aggregation and separation of TM nanorobots, here we first focus on eliminating toxic heavy metals (especially arsenic ions). People are very concerned about the rising level of pollutants, particularly arsenic, in drinking water. These harmful heavy metals can also move through the food chain in vegetables and seafood, causing human diseases and physical, muscular, and neurological degeneration^50,51^. Even at low doses, these materials can cause intense harm to humans and the environment. Therefore, the World Health Organization (WHO) has posted restrictions of arsenic concentrations in drinking water at around 10 µg L^−1^; the WHO also reported in 2001 that about 130 million people worldwide were affected by arsenic concentrations above 25 µg L^−1^^52^.

For the proof of concept, we investigated TM nanorobot performance in low-concentration (25 µg L^−1^) arsenic solution. Following the removal of loaded TM nanorobots from the arsenic-containing water sample, the residual supernatant was examined for arsenic using ICP-OES. The arsenic concentration was not quantifiable within the detection limit of the instrument. This suggests that the TM nanorobots consumed much of the arsenic in the polluted sample. However, when the arsenic disposal from TM nanorobots to water is over 65%, the results presented are averages from the results of three repeated experiments.

**Fig. 5:**
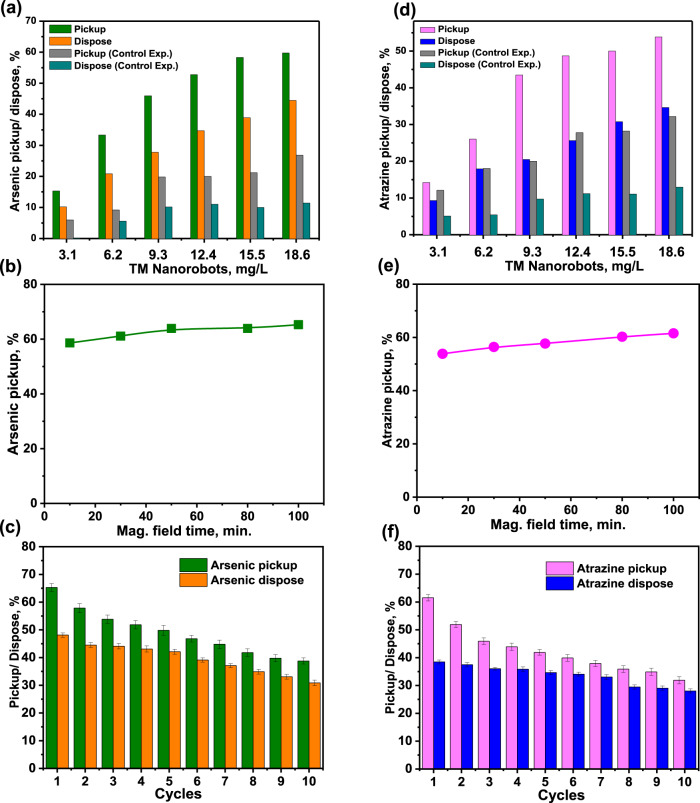
Water remediation by TM nanorobots. (**a**) Influence of TM nanorobots’ adsorbent dosage on pickup and disposal of arsenic (5 mg L^−1^) under a transversal rotating magnetic field (3 mT, 3 Hz for 10 min), whereas control experiment carried out an absence of magnetic field. (**b**) Effect of rotating magnetic-field time on pickup of arsenic by 18.6 mg of TM nanorobots and (**c**) recycling TM nanorobots for pickup and disposal of arsenic under a transversal rotating magnetic field (3 mT, 3 Hz for 100 min). (**d**) Atrazine pickup and disposal as a function of TM nanorobots under a transversal rotating magnetic field (3 mT, 3 Hz for 10 min), whereas control experiments were performed without a magnetic field. (**e**) Influence of rotating magnetic-field time on pickup of atrazine and (**f**) recycling TM nanorobots for pickup and disposal of atrazine under a transversal rotating magnetic field (3 mT, 3 Hz for 100 min). The error bars in recycling TM nanorbots represent standard deviation based on three measurements.

Further examination of the pickup ability of TM nanorobots against toxic arsenic showed dependence upon many interdependent variables such as the concentration of nanorobots, availability of adsorption sites, and adsorption time. Optimization experiments were conducted before the water-treatment studies. Figure 5a analyzes the impact of the amount of TM nanorobots on the elimination of high concentration (5 mg L^−1^) of an arsenic pollutant from a 2 mL solution during 10 min magnetic motion. Parallel control tests were performed to illustrate the impact of TM nanorobots’ movement on the magnetic field. The concentration of arsenic was measured using optical absorption spectroscopy (Supplementary Fig. 4a, b). As seen in Fig. 5a, the amount of arsenic elimination increased from 15.2% to 59.7% upon increasing the amount of TM nanorobots from 3.1 mg to 18.6 mg, respectively. At the same time, arsenic disposal after treatment of TM nanorobots in low-temperature conditions achieved an efficiency of 44.4%. Nevertheless, the same (control) experiment was done without a magnetic field and TM nanorobots were able to pick up only 26.8% of arsenic from the aqueous solution. The microconvection created when TM nanorobots move in the water accelerates the adsorption of contaminants on the TM nanorobots’ surface^53,54^ as this enhances the likelihood of interaction between pollutants and TM nanorobots. Figure 5b shows the effect on remediation time to pick up contaminant over a 10–100 min period using the optimized amount of TM nanorobots (18.6 mg). It was revealed that the arsenic pickup efficiency significantly rises from 59.7 to 65.2% upon increasing the movement time of TM nanorobots from 10 to 100 min, respectively.

Realistic application in water decontamination relying on the adsorption capacity of TM nanorobots, we demonstrate the reuse possibilities of TM nanorobots by modifying the atmospheric temperature. The benefit of a dynamic adsorption system suggested herein is its high stability from the structural and chemical points of view, which provides the opportunity of recycling after every use of the nanorobots by simply pickup and dispose toxic pollutants from them. Figure 5c represents the recyclability of TM nanorobots after the pickup and disposal of arsenic at equilibrium time (100 min). The recycle experiment exhibited arsenic pickup efficiency of the TM nanorobot that dropped from 65.2 to 38%, whereas after ten cooling cycles, the disposal efficiency decreased from 48 to 31%. As shown in Supplementary Fig. 5, a TEM micrograph was employed to evaluate the corrosion or passivation after ten pickup and disposal cycles of TM nanorobots. A uniform PTBC protective film is found on the surface of TM nanorobots. This suggests that Fe_3_O_4_ nanoparticles capped with PTBC have a high level of corrosive resistance. To simulate the ionic strength of media over TM nanorobot propulsion, aggregation, and arsenic removal efficiency, we used 5 mg L^−1^ arsenic solution with different concentrations of sodium chloride (1–100 mM). When the ionic strength was increased, the motion of the TM nanorobots was significantly reduced (Supplementary Fig. 6a, b). However, it does not affect TM nanorobot aggregation. The arsenic pickup rate drastically declines when the ionic strength of the media is increased 100 times (Supplementary Fig. 6c).

In addition to heavy-metal ion pickup and disposal in aqueous media, TM nanorobots can be used for the capture and release of toxic pesticides, especially atrazine, which are highly dangerous to humans. Exposure to atrazine causes serious adverse effects on human health such as ovarian, uterine, breast tumor, as well as lymphoma. It is a damaging endocrine chemical that prevents the routine work of hormones and causes congenital disabilities, reproductive cancers, and significant weight loss in both humans and amphibians^55^. For instance, between 1998 and 2003 in the United States, approximately 7 million individuals were exposed to atrazine in their drinking water^56^. Therefore, the extraction of atrazine from water has become highly necessary.

The influence of TM nanorobot concentration on the pickup of atrazine (5 mg L^−1^) from a contaminated solution is shown in Fig. 5d. A considerably large pickup rate for atrazine, corresponding to the extraction percentage from 14.2% to 53.8%, is observed after 10 min with the amount of TM nanorobots ranging from 3.1 to 18.6 mg, respectively. The concentration of atrazine was measured using optical absorption spectroscopy (Supplementary Fig. 4c, d). The static TM nanorobots (control experiment) achieved only 32.2% atrazine elimination at high concentrations of TM nanorobots. Similarly, the efficiency of atrazine disposal after treatment of TM nanorobots in low-temperature conditions reached 34.6%. Figure 5e demonstrates the correlation between the pickup rate for atrazine and the treatment time using TM nanorobots in a transversal rotating magnetic field. The pickup rate for atrazine grows dramatically when a longer remediation time is used. After around 100 minutes of the transversal rotating magnetic field, 61.5% of atrazine had been extracted. It is worth noting that TM nanorobots show excellent recyclability efficiency to pick up atrazine, which fell from 61.5 to 31.8% after 10 cycles (Fig. 5f). In contrast, the performance of TM nanorobots after 10 cooling cycles, the disposal efficiency decreased from 38.4 to 28%. These experiments were performed using the transversal rotating magnetic field for 100 minutes that is the equilibrium time.

Further maximal pollutant pickup and disposal capability of TM nanorobots at 25 and 5 °C, were investigated. As shown in Supplementary Fig. 7, the pickup and disposal capacity of the TM nanorobots toward both pollutants gradually increases at first and then it exhibits saturation. The fitted model and parameters are depicted in Supplementary Table 1. The estimated maximum pickup and disposal capacity of the Langmuir model was close to the experimental values for both pollutants. The results demonstrate that at 25 °C, the loading capacity of arsenic and atrazine on TM nanorobots is 3.4 and 3.3 mg g^−1^, respectively. However, the disposal capacity of TM nanorobots is 2.3 mg g^−1^ for arsenic and 2.0 mg g^−1^ for atrazine at low temperature (5 °C).

To demonstrate a real practical application of TM nanorobots, we first attempted to pick up high concentrations (100 mg L^−1^) of pollutants using TM nanorobots. The arsenic and atrazine pickup efficiency using 18.6 mg of TM nanorobots was 2.68% and 2.10%, respectively (Supplementary Fig. 8a). These results represent that TM nanorobots removed 2.68 mg L^−1^ and 2.10 mg L^−1^ of arsenic and atrazine, respectively. Moreover, these values are 268- and 700 times higher concentration of the maximum contaminant level of arsenic and atrazine in real water, respectively^52,56,57^. When the TM nanorobot concentration is increased up to 93 mg, then we were able to pick up 11.13% of arsenic and 10.21% of atrazine, representing 1113- and 3403-times higher concentrations of their maximum contaminant levels, respectively. This finding suggests that the TM nanorobots described here might be useful in a variety of real-water cleanup scenarios.

Further, we investigated the remediation capability of TM nanorobots on real wastewater samples using tap and river water as many other pollutants present in a real sample could fill active side groups on the surface of the TM nanorobots. The pickup rates for 5 mg L^−1^ of arsenic and atrazine in tap water were found to be roughly 73% and 72%, respectively, when compared with ultrapure water media (Supplementary Fig. 8b). However, the pickup efficiency of TM nanorobots in river water is 65% for arsenic and 63% for atrazine. Further, the experiment was carried out with ultrapure, tap, and river water containing 1 µM of rhodamine-B dye (model dye) and 5 mg L^−1^ of arsenic and atrazine introduced externally (Supplementary Fig. 8c, d). The TM nanorobots pick up efficiency for arsenic was reduced to less than 10% and atrazine was reduced to 15% when compared with the individual contaminant pickup efficiency (without RB dye). To improve the pickup efficiency of TM nanorobots in realistic water, PTBC with high molecular weight or degree of branching will be of great benefit because more ionic charge could be provided. Therefore, TM nanorobots could be potentially utilized i.e., in water-treatment plants and so on. In these scenarios, we can adjust and monitor temperature for the pickup and disposal of contaminants from water.

Many nanomotors have been described that can efficiently remove heavy-metal ions, accumulate oil droplets, and photodegrade organic pollutants by appropriate structure design and surface modification^25,58^. On the other hand, the fabrication process of these nanomotors is complex and for driving requires a specific wavelength light source, a costly metal catalyst (Pt, Au), and hazardous media (i.e., hydrogen peroxide). They also have weak mechanical properties, such as limited reusability as an absorbent in aqueous media. There are very few reusable nanomotors that have been reported in past years. For instance, V. Singh et al.^59^ employed reusable ZrNPs/graphene/Pt hybrid micromotors for the removal of organophosphate compounds; D. Vilela et al.^60^ reported GOx-microbot-based reusable micromotors for lead-ion decontamination (2-cycle reuse); J. Orozco et al.^61^ used SiO_2_@rGO–Pt Janus magnetic micromotors to remove organic pollutants (4-cycle reuse); and L. Zhang et al.^28^ reported Fe_3_O_4_ nanoparticles for dye degradation with 73.5% retained rate after three cycles. In these studies, the majority of nano-/micromotors were recycled in the presence of an acidic medium or organic solvent (e.g., isooctane, ethanol), among other desorption methods^59–61^. Such harmful solvents would be detrimental to the active surface of nano/micromotors and cause a decrease of their effective lifespan for contaminant adsorption.

Here we present a TM nanorobot fabrication process that is very simple and utilizes components that are fully biocompatible. TM nanorobots can easily pick up and dispose of contaminants by altering the temperature of the surrounding media. They can also be reused after a simple cooling process. This study creates new approaches for fabricating various surface-tunable magnetite nanorobots to address new environmental remediation needs.

In summary, temperature-responsive nanorobots have been reported for the efficient pickup and disposal of arsenic and atrazine from contaminated water by adjusting the temperature. Synthesis of TM nanorobots was performed using facile chemical methods and equipment, which is a significant benefit to future large-scale manufacturing. The mechanism of pickup and disposal of toxic pollutants was observed by the aggregation and separation of TM nanorobots at room and low temperatures, respectively. The magnetic properties and temperature-sensitive phase transferability of the TM nanorobots were utilized to recover nanorobots and toxic contaminants from the water after the remediation process, respectively. The extraction and recovery of toxic pollutants were successfully performed for ten cycles. In contrast to typical nanomotors, this design could be utilized to adjust the surface property of the TM nanorobots by changing the type of functional groups (e.g., -OH, -NH_2_, and -COOH) according to practical needs. Also, TM nanorobots containing PTBC functionalized with a large molecular weight or degree of branching will greatly aid in improving the surface charge, which will boost the pollutant removal efficiency of TM nanorobots. It can be produced on a large scale due to the low cost and good biodegradability of PTBC and Fe_3_O_4_. This technique presents an eco-friendly and affordable alternative to other kinds of thermoresponsive nanorobots for various environmental remediation applications.

## Methods

### Materials and reagents

Ferric chloride hexahydrate (FeCl_3_ 6H_2_O), ammonium hydroxide (NH_4_OH), ferrous chloride tetrahydrate (FeCl_2_ 4H_2_O), pluronic triblock copolymer (PTBC), rhodamine B dye, and oleic acid were purchased from Sigma-Aldrich and used as received. Ultrapure water was utilized for the preparation and application of TM nanorobots. The natural river water was collected from the Vltava River in the section located on Kralupy nad Vltavou city, Czech Republic.

### Synthesis of TM nanorobots

Fe_3_O_4_ nanoparticles were synthesized by FeCl_3_ 6H_2_O and FeCl_2_ 4H_2_O with NH_4_OH. About 3 mL of 5 M NH_4_OH was dropped into an equimolar solution of FeCl_3_ 6H_2_O (15 mL) and FeCl_2_ 4H_2_O (7.5 mL) under continuous stirring. The entire mixture was stirred for 15 min at ambient temperature and then filled with 100 mg of oleic acid. After heating the mixture to 80 °C for 5 h, it was cooled to room temperature. Oleic acid-functionalized Fe_3_O_4_ nanoparticles were isolated by sticking a neodymium magnet below the glass container and removing the subsequent supernatant. The separated oleic acid-functionalized Fe_3_O_4_ nanoparticles were washed several times with water and dispersed by sonication. Afterward, oleic acid-decorated Fe_3_O_4_ nanoparticles were introduced to 0.1 g of PTBC and the particle suspension was stirred overnight. Finally, the TM nanorobot suspension was washed with water using magnetic separation and used for further experiments.

### Characterization and motion evaluation of TM nanorobots

Fourier transform-infrared (FTIR) spectroscopy was carried out using Nicolet 6700 FTIR spectrometer (Thermo-Nicolet, USA) in conjunction with a GladiATR diamond ATR attachment (PIKE, USA), reflectance detector, and DTGS KBr detector. Samples were scanned in the range of 4000–400 cm^−1^, resolution 4 cm^−1^, number of spectra accumulations 64, and Happ–Genzel apodization. The XRD analysis of pure Fe_3_O_4_ nanoparticles and TM nanorobots was performed with a PANalytical X´Pert PRO (The Netherlands). The parameters chosen for the measurement were 2θ range from 20° to 70° with steps of 0.039°. UV–visible spectrophotometric (UV-2450, SHIMADZU) analysis was performed to confirm quantitatively measured pollutant content in water. Inductively coupled plasma optical emission spectrometry (ICP-OES, Spectro Arcos), was used for the determination of arsenic. Motion videos and optical microscopy images of the TM nanorobots in the different magnetic fields were recorded by an inverted optical microscope Olympus (CKX53) coupled with a Photron Mini AX200 high-speed camera and Basler acA1920-150uc camera. Transversal rotating magnetic field was generated by home-made triaxial coils. This coils system was integrated with the inverted optical microscope. TEM micrographs were obtained for the TM nanorobots using an EFTEM Jeol 2200 FS microscope with an accelerating voltage of 200 kV.

### Pollutant removal and recovery experiments

To analyze the adsorption isotherms of toxic pollutants in the TM nanorobots, varying amounts of TM nanorobots (3.1–18.6 mg) were added to 2 mL of arsenic (5 mg L^−1^) and atrazine (5 mg L^−1^) solutions that were previously cooling to 5 °C. The entire mixture was placed inside six triaxial coils and a transversal rotating magnetic field with a constant frequency of 3 Hz and magnetic-field intensity of 3 mT was applied over 10 min at ambient temperature. Afterward, TM nanorobots were isolated using a niobium magnet and the resultant supernatant was measured for pollutants by UV–visible spectroscopy.

The pollutant loading capacity at equilibrium (*q*_e_) and pickup efficiency on TM nanorobots were calculated by Eq. (1) and (2)^62^:

1 $${q}_{e}=\frac{{C}_{0}-{C}_{e}}{m}\times V,\ldots \ldots \ldots .$$

2 $$\% \,\, {{{{{\rm{Pickup}}}}}}\; {{{{{\rm{efficiency}}}}}}=\frac{{C}_{0}-{C}_{e}}{{C}_{0}}\times 100,\ldots \ldots \ldots .$$

where C_0_ is the initial concentration in solution (mg/L), *C*_*e*_ is the equilibrium concentration after pickup (mg/L), V represents the volume of solution (L), and m is the mass of TM nanorobots. Similarly, optimized amounts of TM nanorobots were used with a different remediation time range of 10–100 min and the impact on pollutant removal efficiency was observed. For the disposal of absorbed contaminants from the surface of TM nanorobots, these were mixed in 2 mL of ultrapure water in a glass bottle and kept below 5 °C to detach pollutants from the surface of TM nanorobots. The pollutant disposal capacity at equilibrium (*q*_de_) was calculated by Eq. (3). The pollutant desorption was expressed as a disposal efficiency (%). It was calculated by the amount of released contaminant over total contaminant absorbed on TM nanorobots. Disposal efficiency was calculated using Eq. (4):

3 $${q}_{{{{{\rm{de}}}}}}=\frac{{C}_{1}\times V}{m},\ldots \ldots \ldots .$$

4 $$\% \,\, {{{{{\rm{Disposal}}}}}}\; {{{{{\rm{efficiency}}}}}}=\frac{{q}_{{{{{\rm{de}}}}}}}{{q}_{e}}\times 100,\ldots \ldots \ldots .$$

where *C*_1_ represents the concentration after release of adsorbed pollutants from a surface of TM nanorobots. Experimental results were examined with reference to Langmuir isotherm (Eq. (5))^62,63^

5 $${q}_{e}=\frac{{q}_{m}{K}_{L}{C}_{e}}{1+{K}_{L}{C}_{e}}\ldots \ldots \ldots .$$

where K_L_ (L/mg) represent the Langmuir constant that shows the binding affinity between pollutants and TM nanorobots and *q*_m_ (mg/g) define the maximum loading capacity of TM nanorobots. Afterward, the TM nanorobots were separated by the magnet and rinsed with water for reuse. A low concentration (>25 µg L^−1^) of arsenic in water was quantified by ICP-OES analysis. All pickup and disposal experiments were conducted three times. In real samples, river water was contaminated with many different types of microplastic and microorganisms. Therefore, the river water was filtered and diluted with distilled water in a 1:1 ratio. Similarly, tap water was diluted in a 1:1 ratio with distilled water before usage. To prepare complex samples, rhodamine-B dye stock solution was made by dissolving an adequate quantity of dye powder in ultrapure, tap, and river water, and a lower dye concentration (1 μM) was obtained by diluting the stock solution.

## Supplementary information

Supplementary Information

Description of Additional Supplementary Files

Supplementary Movie 1

## Data Availability

The data generated in this study are provided in the paper or its supplementary Information and FigShare repository (10.6084/m9.figshare.17278619).
