# Supplementary material for: Pick up and dispose of pollutants from water via temperature-responsive micellar copolymers on magnetite nanorobots
Source: Nat Commun. 2022 Mar 1;13:1026. doi: 10.1038/s41467-022-28406-5 (PMC8888651; doi:10.1038/s41467-022-28406-5)
Supplement: Supplementary file 1 — Supplementary Information [file 41467_2022_28406_MOESM1_ESM.pdf]

Supplementary Information

Pick up and dispose of pollutants from water via temperature-responsive micellar copolymers on magnetite nanorobots

Jayraj V. Vaghasiya,[1] Carmen C. Mayorga-Martinez,[1] Stanislava Matějková,[2] Martin Pumera[1,3,4,5,6*]

[1]Center for Advanced Functional Nanorobots, Department of Inorganic Chemistry, Faculty of Chemical Technology, University of Chemistry and Technology Prague, Technická 5, 166 28 Prague 6, Czech Republic
[2]Central Analytical Laboratory, Institute of Organic Chemistry and Biochemistry of the Academy of Sciences of the Czech Republic, 166 10 Prague 6, Czech Republic
[3]Department of Chemical and Biomolecular Engineering, Yonsei University, 50 Yonseiro, Seodaemun-gu, Seoul 03722, Korea
[4]Department of Medical Research, China Medical University Hospital, China Medical University, No. 91 Hsueh-Shih Road, Taichung 40402, Taiwan
[5]Center for Nanorobotics and Machine Intelligence, Dept. of Food Technology, Mendel University, Zemedelska 1, Brno 613 00, Czech Republic
[6]Future Energy and Innovation Lab, Central European Institute of Technology, Brno University of Technology, Purkyňova 123, Brno 612 00, Czech Republic

E-mail: pumera.research@gmail.com

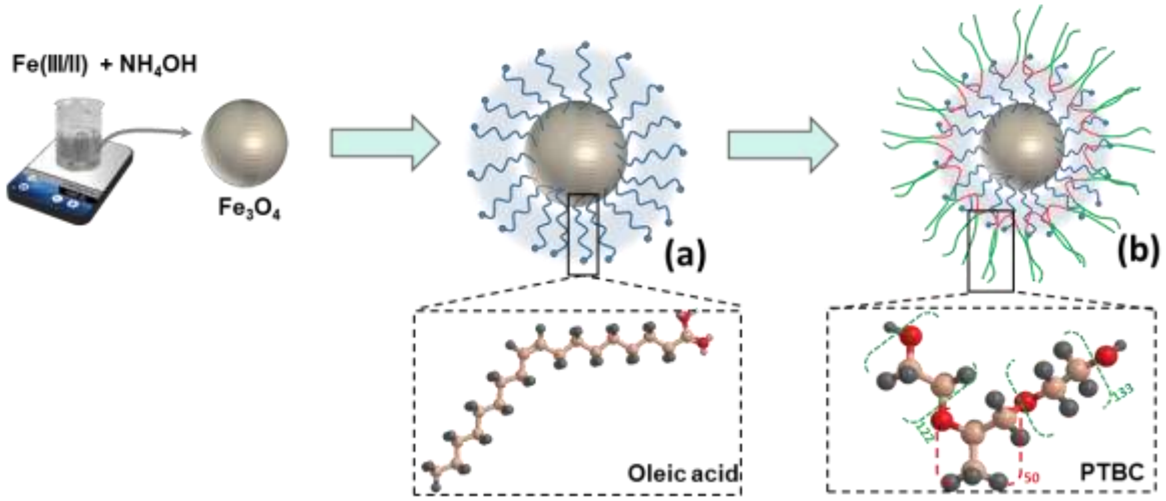

Supplementary Figure 1: Synthesis of TM nanorobots. (a) oleic acid functionalization on Fe_3O_4 nanoparticles and (b) PTBC decorated on oleic acid@Fe_3O_4 nanoparticles.

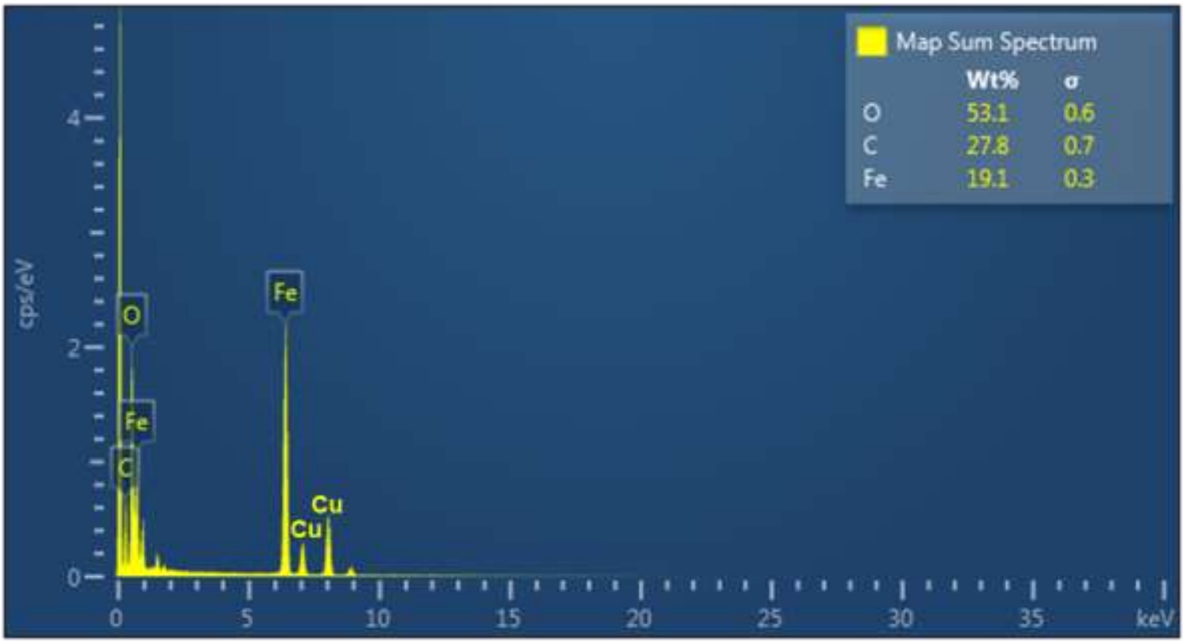

Supplementary Figure 2: EDS spectrum of TM nanorobots. Note that the copper peak in the spectrum arises from the sample support STEM grid.

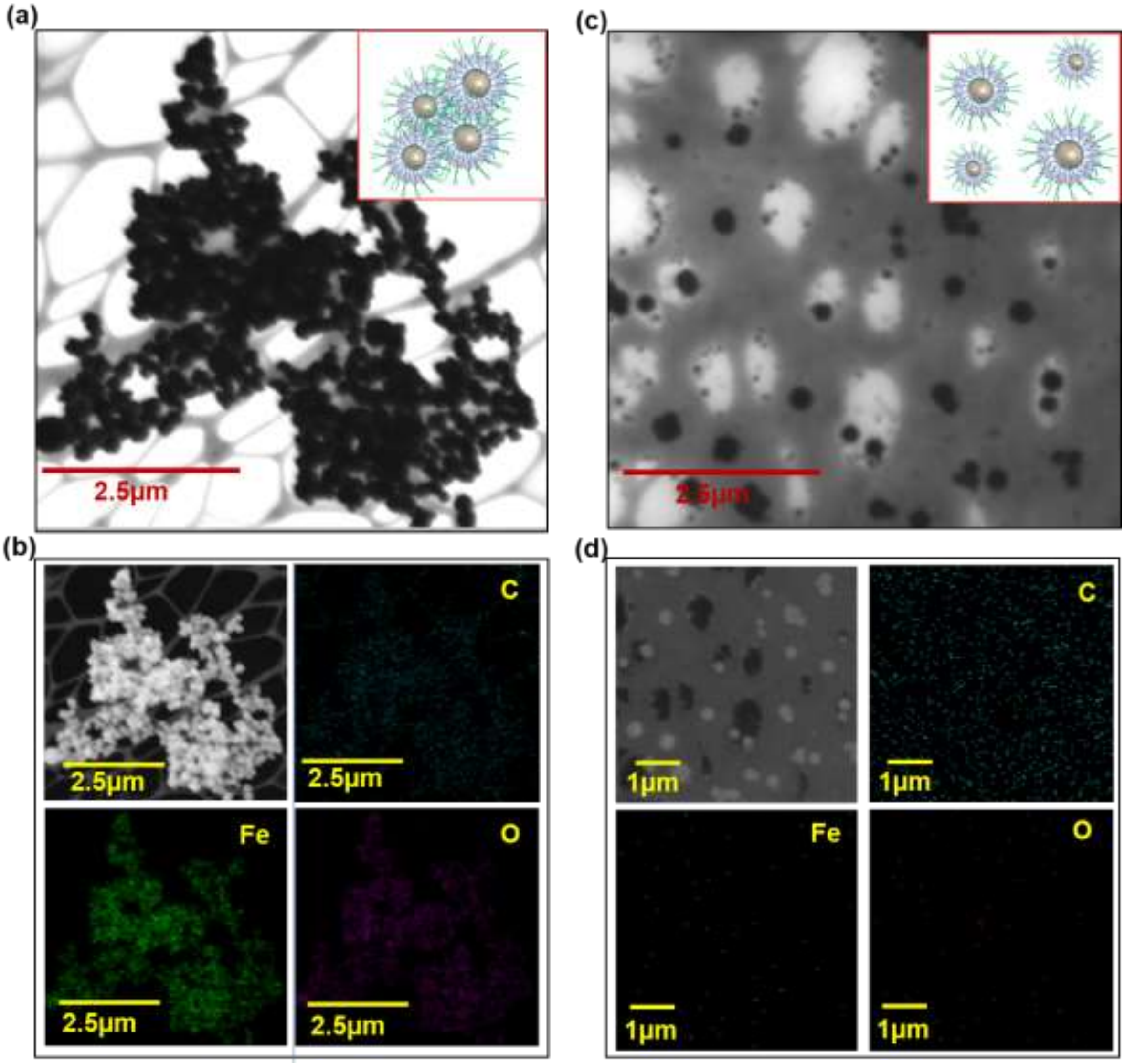

Supplementary Figure 3: TM nanorobot morphology study as a function of temperatures; (a, b) STEM image, EDS mapping of TM nanorobots prepared at 25 ºC, (c, d) STEM image, EDS mapping of TM nanorobots prepared at 5 ºC.

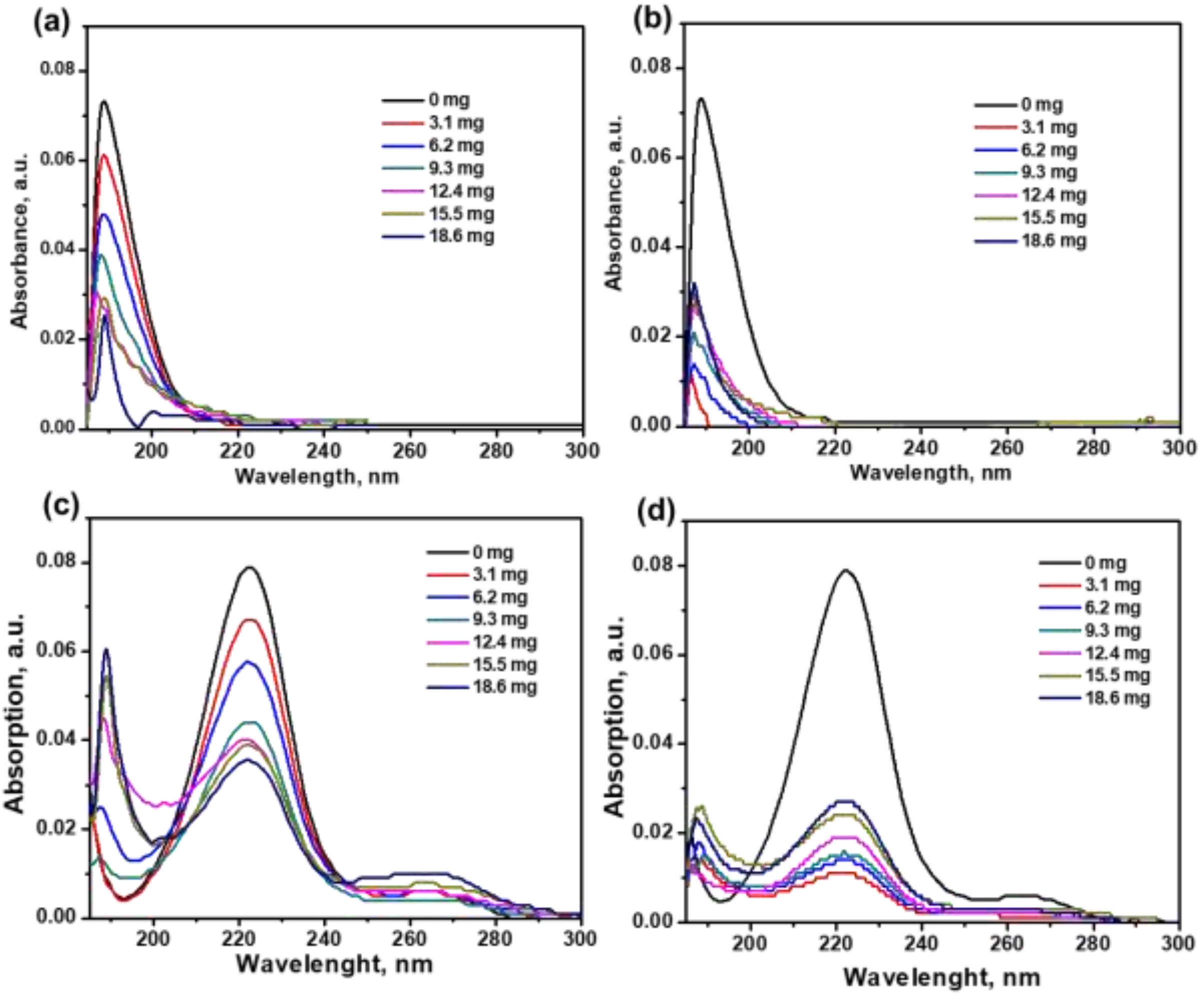

Supplementary Figure 4: Absorbance spectrum of pollutants. (a) Pickup and (b) dispose arsenic ions at different concentration of TM nanorobots. (c) Pickup and (d) dispose atrazine at different concentration of TM nanorobots.

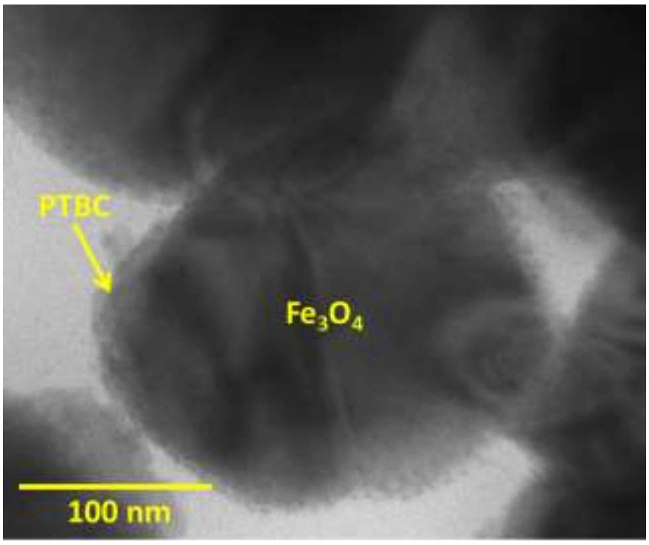

Supplementary Figure 5: TEM image after ten cycles of arsenic removal by TM nanorobots.

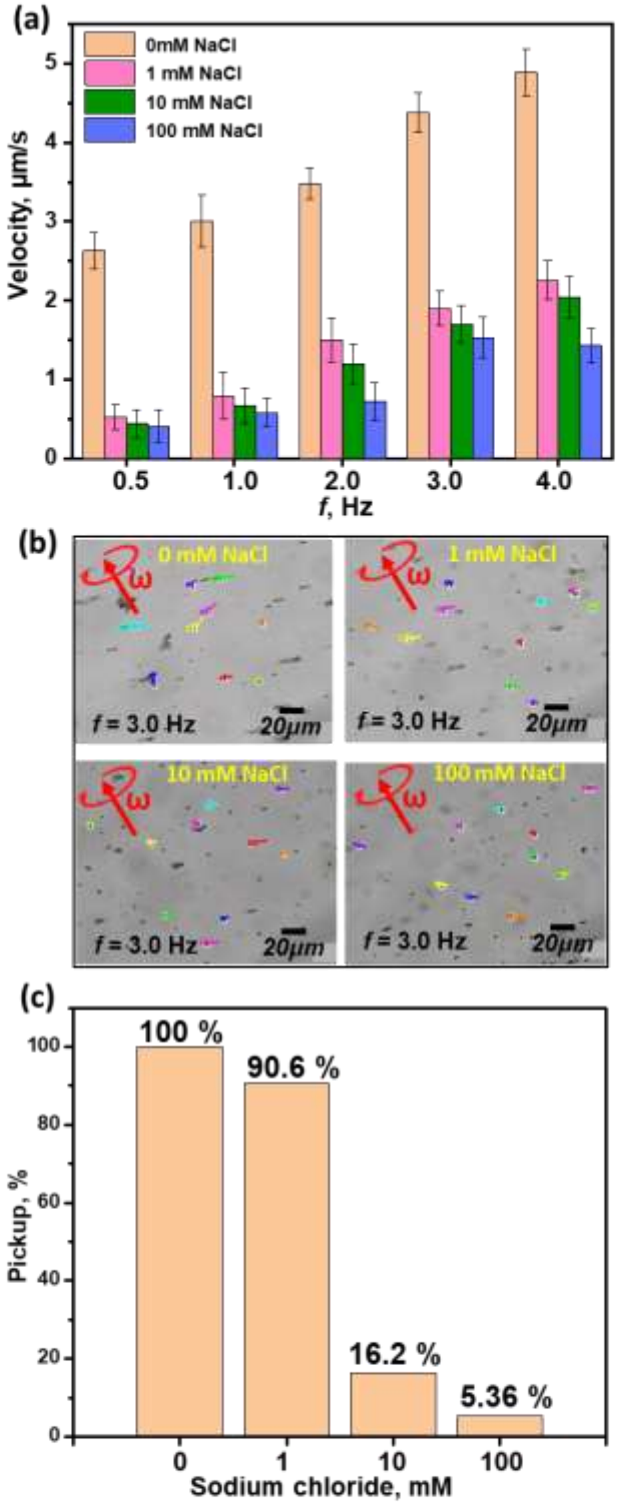

Supplementary Figure 6: Ionic strength study at varying sodium chloride solution (1mM to 100mM); (a) plot of velocity versus different frequency. Error bars in the velocity of TM nanorbots indicate the standard deviation obtained from three repeated experiments, (b) tracking optical images and (c) removal efficiency (note: different concentration of sodium chloride solution prepared in 5ppm arsenic).

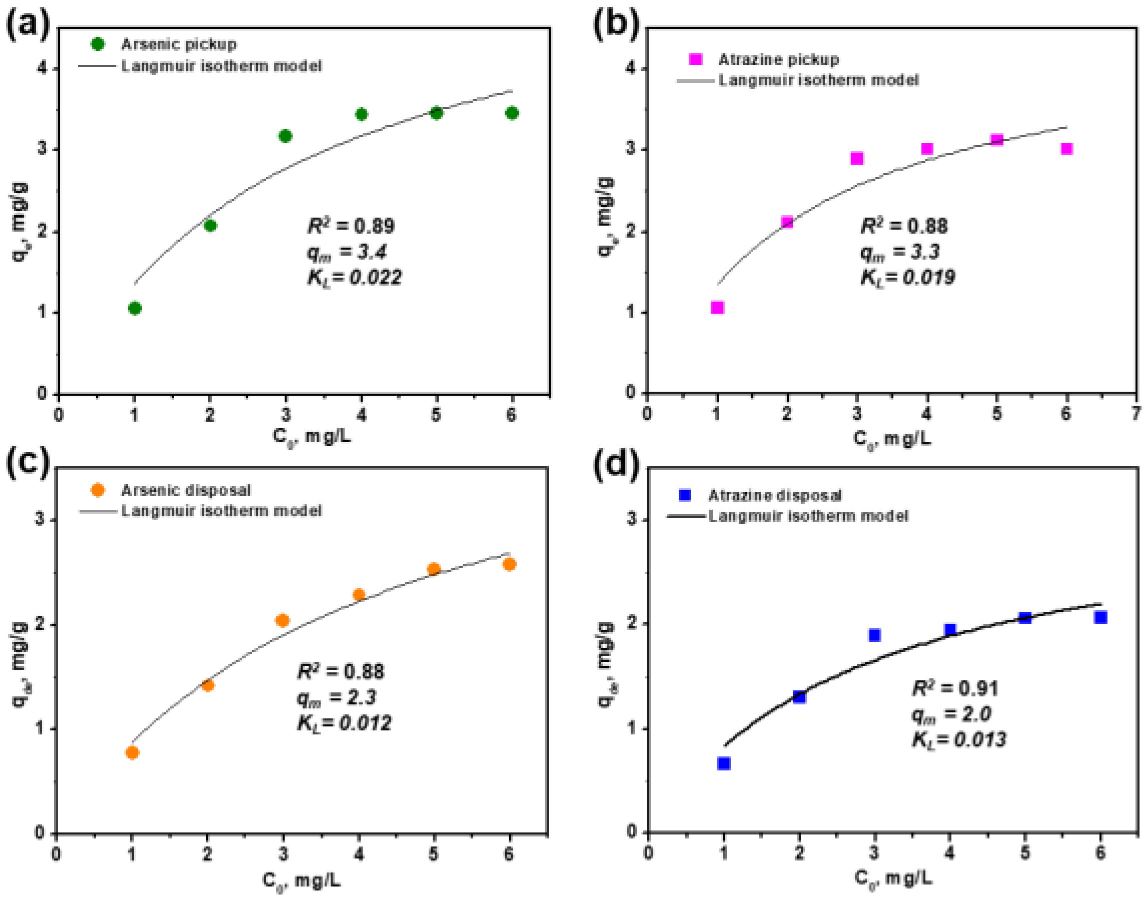

Supplementary Figure 7: Langmuir isotherms for pollutants pickup at 25 ºC (a-b) and disposal at 5 ºC (c-d) by TM nanorobots. [Experimental conditions: TM nanorobots dosage = 0.0186 g L^{-1}; magnetic field = 3 mT. 3Hz; contact time = 100 min].

Supplementary Table 1: Physico-chemical parameters for the pickup/disposal of pollutants by TM nanorobots, based on the data shown in Supplementary Figure 7 and Eq. 5.

Fitting parameters	Arsenic		Atrazine	
	at 25 °C	at 5 °C	at 25 °C	at 5 °C
R^2	0.89	0.88	0.88	0.91
q_m (mg/g)	3.4	2.3	3.3	2.0
K_L (L/mg)	0.022	0.012	0.019	0.013

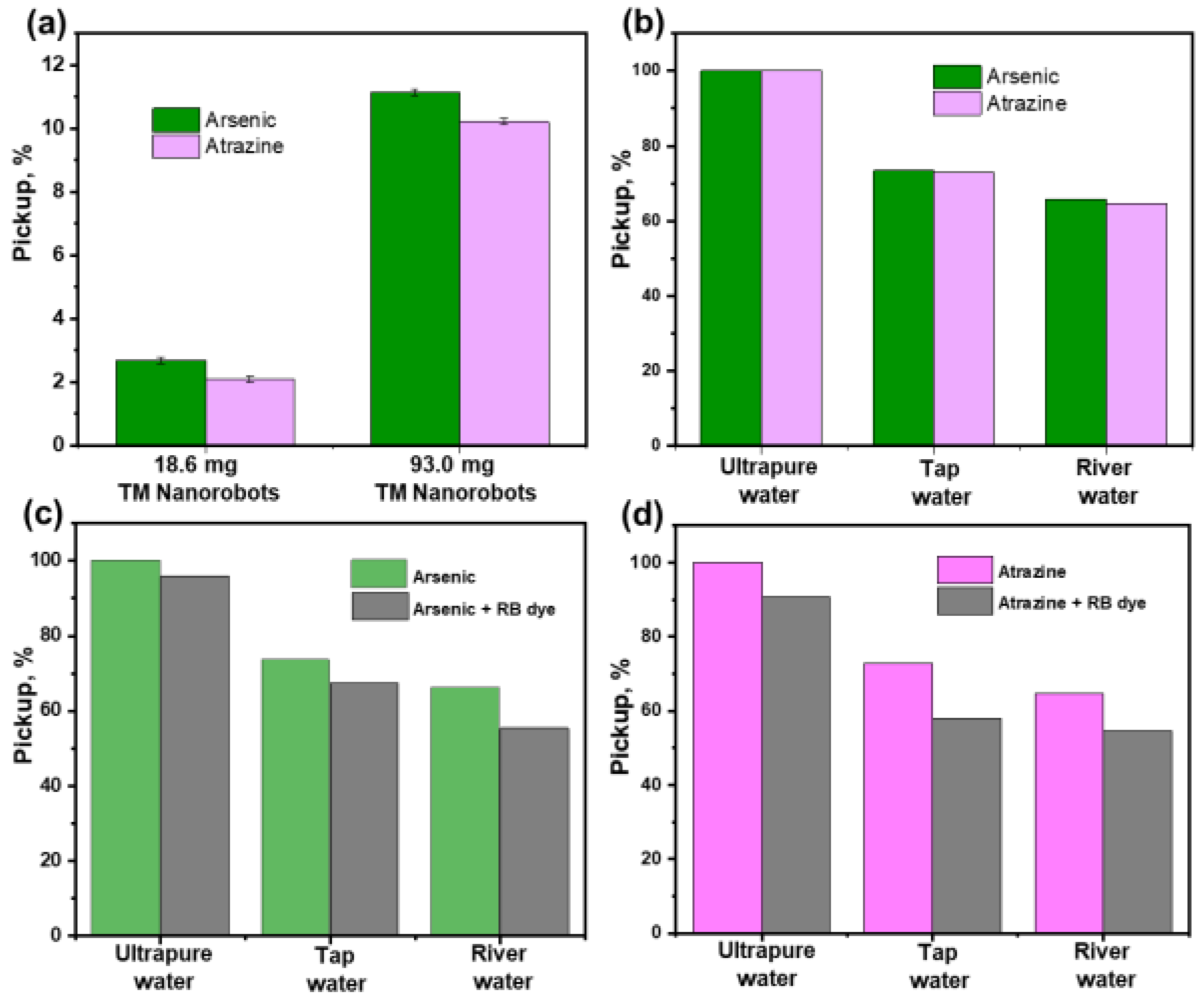

Supplementary Figure 8: TM nanorobots efficiency in real polluted waters. (a) High concentration (100 mg L^{-1}) of pollutant pickup by TM nanorobots. The error bars represent standard deviation based on three measurements; (b) TM nanorobot pickup efficiency in ultrapure, tap and river water samples; (c) efficiency of TM nanorobots to pick up arsenic from realistic water samples with and without rhodamine B (RB) dye; (d) efficiency of TM nanorobots to pick up atrazine from realistic water samples with and without rhodamine B (RB) dye.
