# Supplementary material for: Pick up and dispose of pollutants from water via temperature-responsive micellar copolymers on magnetite nanorobots
Source: Nat Commun. 2022 Mar 1;13:1026. doi: 10.1038/s41467-022-28406-5 (PMC8888651; doi:10.1038/s41467-022-28406-5)
Supplement: Supplementary file 2 — Description of Additional Supplementary Files [file 41467_2022_28406_MOESM2_ESM.pdf]

Description of Additional Supplementary Files

Supplementary Movie 1. Shows the (a) toxic pollutants payload onto TM nanorobots and begin to aggregate, (b) TM nanorobots' intermicellar aggregation at room temperature, and (c) toxic pollutant dispose and dispersion of TM nanorobots at low temperature.
